# Highly Nanoporous Nickel Foam as Current Collectors in 3D All-Solid-State Microsupercapacitors

**DOI:** 10.1021/acsomega.4c05514

**Published:** 2024-08-20

**Authors:** Bayu Satriya Wardhana, Kuan-Wen Wang, Wei-Hsuan Hung, I-Yu Tsao, Pin-Ching Chen, Jason Shian-Ching Jang, Shih-Chieh Hsu, Sheng-Wei Lee

**Affiliations:** † Institute of Materials Science and Engineering, National Central University, Taoyuan City 32001, Taiwan, ROC; ‡ Department of Mechanical Engineering, Brawijaya University, Malang City 65145, Indonesia; § Department of Mechanical Engineering, National Central University, Taoyuan City 32001, Taiwan, ROC; ∥ Department of Chemical and Materials Engineering, Tamkang University, New Taipei City 25137, Taiwan, ROC; ⊥ Department of Chemical and Materials Engineering, National Central University, Taoyuan City 32001, Taiwan, ROC; # Graduate College of Sustainability and Green Energy, National Central University, Taoyuan City 32001, Taiwan, ROC

## Abstract

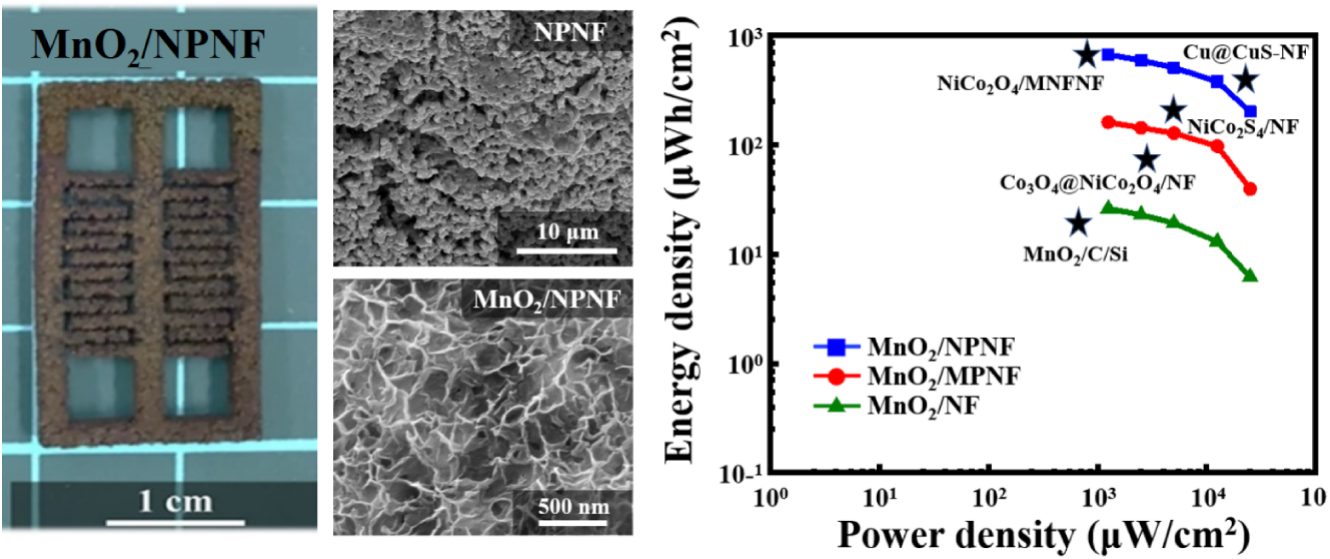

This study reports a streamlined method for producing a highly nanoporous current collector with a substantial specific surface area, serving as an electrode for microsupercapacitors (MSCs). Initially, commercial Ni foams are patterned into an interdigitated structure by laser cutting. Subsequently, the Ni foams are infused with NiO nanopowders through dip coating, sintering, and reduction in an H_2_ atmosphere, followed by the growth of MnO_2_ through a redox reaction. The incorporation of NiO within this three-dimensional Ni current collector results in notable porosity within the range of approximately 200–600 nm. Such a 3D, highly nanoporous electrode dramatically increases the specific surface area by 30 times and substantially boosts the amount of active material deposition, surpassing those of commercially available Ni foams. Performance evaluations of this highly nanoporous electrode in a 1 M KOH solution demonstrate an areal capacity of 19.3 F/cm^2^, retaining more than 95% capacitance at 5 mA/cm^2^, and exhibiting an energy density of 671 μW h/cm^2^, 25 times greater than commercial Ni foams. Moreover, in the realm of solid-state applications for MSCs, the remarkably high porous electrode achieves a commendable areal capacity of 7.22 F/cm^2^ and an energy density of 263.9 μW h/cm^2^, rendering it exceptionally suitable for use in MSC applications.

## 1 Introduction

Microsupercapacitors (MSCs) stand as the primary choice among cutting-edge miniature energy storage devices, owing to their impressive traits: high power density, extended lifespans, and rapid charge/discharge rates.^1−3^ However, when compared to Li-ion-based microbatteries, MSCs exhibit a lower energy density. Consequently, enhancing the volumetric or area energy density becomes imperative for tackling demanding applications.^4,5^ Numerous endeavors have been undertaken to elevate electrochemical performance, chiefly through the advancement of highly active electrode materials.^6,7^ An ideal electrode material necessitates distinct characteristics, including excellent conductivity, a substantial active surface area, and efficient ion diffusion pathways.^8−10^ Furthermore, optimizing the microstructure of electrodes emerges as a pivotal avenue to amplify MSC performance.^11−14^ The structural designs of MSCs commonly fall into categories such as conventional sandwiches, rolls, and interdigitated structures.^15,16^ Among these structures, the interdigitated configuration has garnered significant attention due to its narrow gap structure, presenting various advantages over conventional designs. These advantages encompass low charging current requirements, mitigated solution resistance effects, and a current flow regulated by diffusion.^17,18^

Within the manufacturing process of MSC electrodes, two distinct approaches facilitate the realization of the interdigitated electrode design. The first approach termed the bottom-up method,^19^ involves the integration or construction of electrodes from small to microsized powders or particles. These particles are molded into a dense paste or colloidal suspension,^20^ and subsequently shaped via techniques such as inkjet printing,^16,21,22^ screen printing,^23−25^ or electrophoretic deposition.^26,27^ In contrast, the top-down approach employs in situ processing or synthesis of active components,^20^ encompassing methodologies like plasma etching,^28^ direct laser writing,^29−31^ or photolithographic techniques.^32^ Notably, laser-based techniques possess several inherent advantages, including their high precision and efficiency, rendering them among the most extensively utilized methods in crafting interdigitated structure electrodes.^18,31,33^ The versatility of lasers has been underscored in various studies, showing their remarkable potential in manufacturing micro/nanostructures,^34^ and extending to the fabrication of three-dimensional (3D) porous structures in current collectors.^35,36^

The utilization of 3D porous structures in MSCs has gained widespread recognition due to their ability to enhance the mass loading of active materials, expedite mass transfer kinetics, and shorten ion-electron diffusion pathways.^37^ Among the extensively explored 3D porous materials, Ni foam (NF) has found application as a current collector.^38−40^ Typically characterized by a porosity range of 70–90% per unit area or volume, NF exhibits pore sizes spanning from a few micrometers to millimeters. However, further improvements are necessary for it to serve as an exemplary current collector.^35,41^ Several methods have been explored to optimize large porous NF, aiming to accommodate substantial mass loading of active ingredients without the use of binding agents,^6,42,43^ Yu et al. introduced an intriguing approach by creating a Ni-filled NF micro/nano current collector. This involved filling commercial Ni foam with Ni slurry and sintering it to form micro Ni-filled Ni foam, followed by electrochemical deposition of nano Ni. The addition of Ni to commercial nickel foam resulted in increased surface area, elevated mass loading of active material, and heightened areal capacitance.^40^ However, during sintering, the Ni slurry tends to agglomerate easily, leading to the degradation of porous microstructures.

Therefore, the appropriate modification of commercial NF frameworks significantly influences the specific surface area and electrochemical performance of various electrodes in MSC applications.^40,44^ This study focused on improving NF by infusing it with NiO powders, followed by a process involving sintering, reduction, and eventually growth of the active material MnO_2_. In contrast to Ni, which tends to agglomerate during sintering,^45^ NiO exhibits much slower self-diffusion and thus a better ability to resist agglomeration.^46^ The slower self-diffusion of NiO restrains neck growth and densification, consequently impacting the final pore size and porosity of the Ni electrode. Finally, MnO_2_ was chosen as the active material for MSCs in this work, due to its high theoretical capacitance, cost-effectiveness, and environmental safety.^47,48^ The amalgamation of highly porous NF electrodes with these active materials aims to yield a 3D current collector that exhibits superior and competitive performance.

## 2 Experimental Section

### 2.1 Interdigitated Electrode Manufacturing

**Figure 1:**
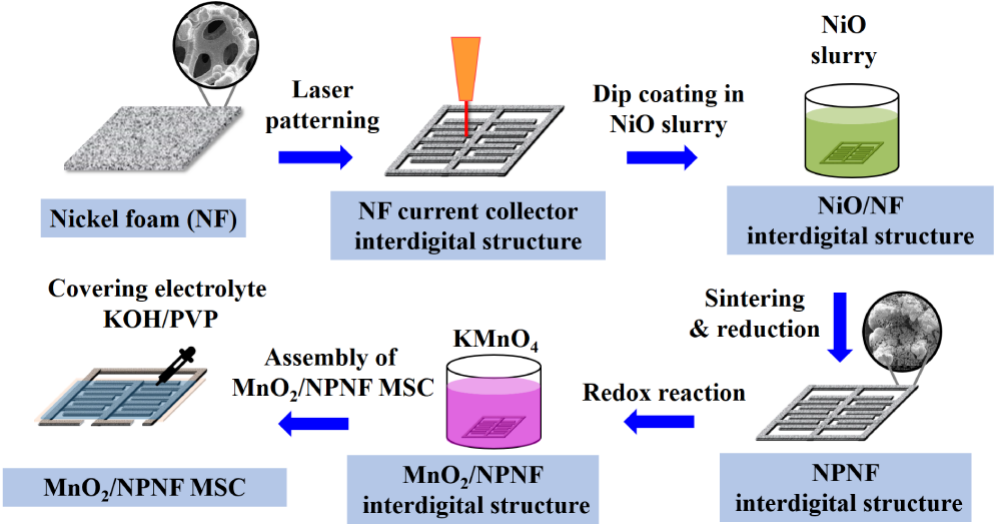
Process flow illustrating the fabrication of an all-solid-state MSC utilizing the interdigitated NPNF electrodes.

**Figure 2:**
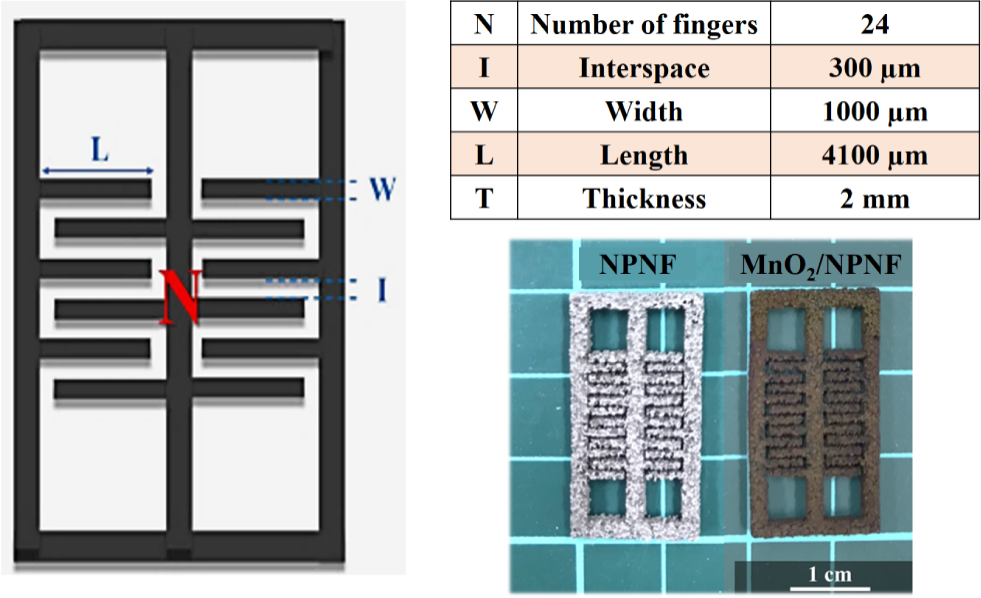
Geometrical structural parameters and photographs of the interdigital NPNF structure.

Figure 1 depicts a schematic overview of the MSC manufacturing process. Initially, a 2 mm thick commercial NF was patterned to the interdigitated shape using a high-power pulsed laser, The geometric structural specifications of the interdigitated structure are depicted in Figure 2. The laser parameters used here were a wavelength of 1064 nm, a frequency of 80 kHz, and a spot size of 100 μm. With a peak laser power of 40 W and a scanning rate of 10 mm/s, the NF can be precisely patterned to achieve the desired structure. After ultrasonic cleaning in deionized water and ethanol, the interdigitated NF underwent a dip coating process, utilizing a slurry of commercial NiO (Showa, 99.8%) in a mixture of PVP and ethanol at a ratio of 52:6:42, respectively. Following the drying of the NiO slurry, sintering was carried out in an atmospheric environment at 1000 °C for 2 h, succeeded by a reduction in an H_2_ environment at 600 °C. The highly nanoporous interdigitated NF was denoted as NPNF in the subsequent discussion. For reference, the interdigitated NF was similarly dip-coated using a slurry of Ni nanopowders (Gredmann, 99.9%) with an identical chemical ratio. The Ni-filled NF underwent a similar sintering and reduction process, exhibiting microporous microstructures, and was consequently labeled as MPNF. The active material, MnO_2_, was then deposited on the interdigital NF, NPNF, and MPNF electrodes through a chemical redox reaction.^49^ A solution of 0.01 M KMnO_4_ in deionized water serves as the precursor, immersing the interdigital NF-based current collectors for a duration of 30 h at a temperature of 90 °C to form MnO_2_, as shown in the photographs of Figure 2. The growth of the MnO_2_ structure over the NF-based current collectors follows the chemical reaction below (eq 1):^50^
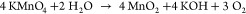
1

After being dried in an oven, the interdigital NF-based structure was precisely cut into two electrodes before being assembled into MSCs. To produce the solid electrolyte, a solution was prepared by combining 10 g of PVP in 40 mL of deionized water alongside another solution comprising 2.8 g of KOH in 10 mL of deionized water. These solutions were thoroughly mixed and stirred until a homogeneous gel of 1 M KOH was obtained. Subsequently, the gel was applied as a solid electrolyte coating onto the electrodes.

### 2.2 Material Characterizations and Electrochemical Testing

The X-ray diffraction was applied to the crystallographic analysis of MnO_2_-coated NF-based electrodes, where the diffractometer (XRD, Bruker D2 Phaser) operated at 30 kV and 20 mA using Cu Kα radiation with a scan rate of 0.04° per step from 2θ = 10° to 80°. Field-emission scanning electron microscopy (FE-SEM, FEI Quanta 200F) was employed to characterize the electrode microstructures. Various electrochemical tests including cyclic voltammetry (CV), galvanostatic charge–discharge (GCD), and electrochemical impedance spectroscopy (EIS) were carried out using an electrochemical workstation BioLogic SP-150 with a 1 M KOH electrolyte to gather the results. In this study, electrochemical testing was performed by using a three-electrode system. The interdigital NF-based structures functioned as the working electrode, while reference and counter electrodes comprised Ag/AgCl and Pt foil, respectively. The areal capacity was calculated according to the following equation (eq 2):
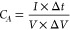
2where CA = areal capacitance (F/cm^2^), *I* = discharging current density (A/cm^2^) that is corresponding to the geometric area,  = discharging time (s), Δ*V* = potential window range (*V*), and *A* = geometric electrode area (cm^2^).^40^

Meanwhile, the energy density (*E*) and power density (*P*) were calculated using the following equations (3 and 4), respectively:
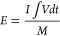
3and
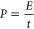
4where *M* = total mass loading of active material (g) and  is the galvanostatic discharge current area.^51^

## 3 Results and Discussion

### 3.1 Material Characterizations

**Figure 3:**
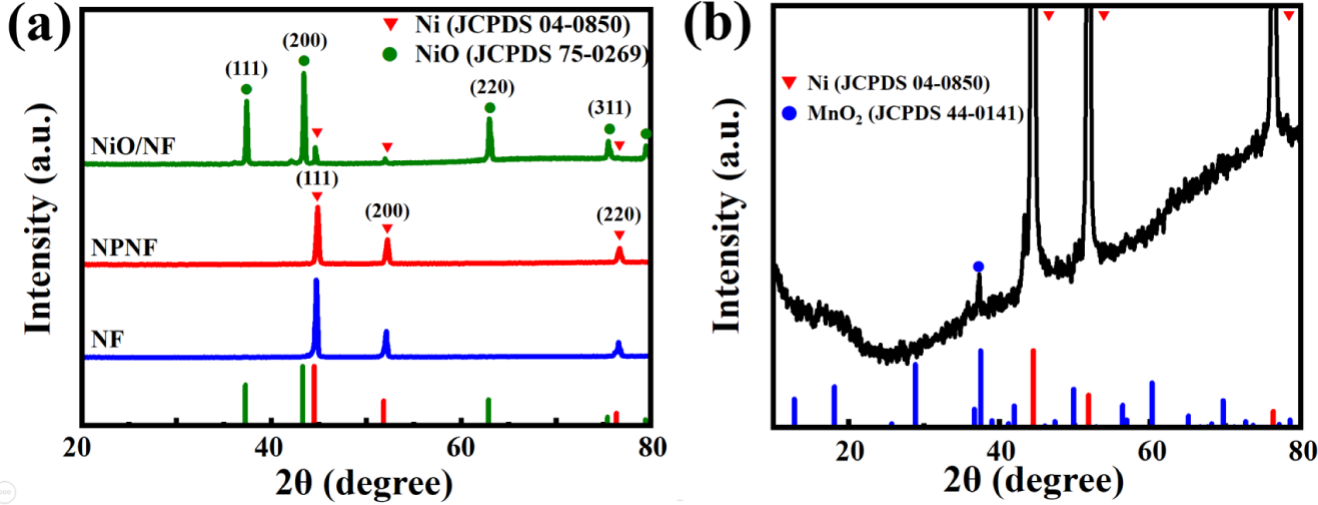
XRD patterns of (a) the NiO-filled NF, NPNF, and commercial NF current collectors and (b) MnO_2_-coated NPNF electrode.

Figure 3a illustrates the XRD patterns of NiO-filled NF, NPNF, and commercial NF current collectors. In the NiO-filled NF sample, peaks at approximately 44°, 52°, and 76° correspond to (111), (200), and (220) planes of Ni phases, respectively (JCPDS card no. 04-0850).^52^ Distinct diffraction peaks around 37°, 43°, 62°, and 75° indicate the presence of NiO within NF (JCPDS card no. 75-0629).^53^ After a reduction in an H_2_ environment, the diffraction pattern exclusively shows pure Ni (NPNF), suggesting the transformation of NiO into a completely pure Ni phase at the NPNF current collector. Moving to Figure 3b, the diffraction pattern of the MnO_2_/NPNF electrode reveals a blend of amorphous and crystalline structures. Ni-related peaks remain obvious, while MnO_2_ is only detected at around 37.5° (JCPDS-44-0141),^41^ suggesting that the MnO_2_ layer on the NPNF electrode surface is so thin that it lacks the strength to produce observable peaks in XRD patterns. Raman spectroscopy was further used to verify the MnO_2_ grown on the electrodes. As shown in Figure S1, Raman shifts around 480 and 591 cm^–1^ can be attributed to the presence of MnO_2_,^6,49,54^

**Figure 4:**
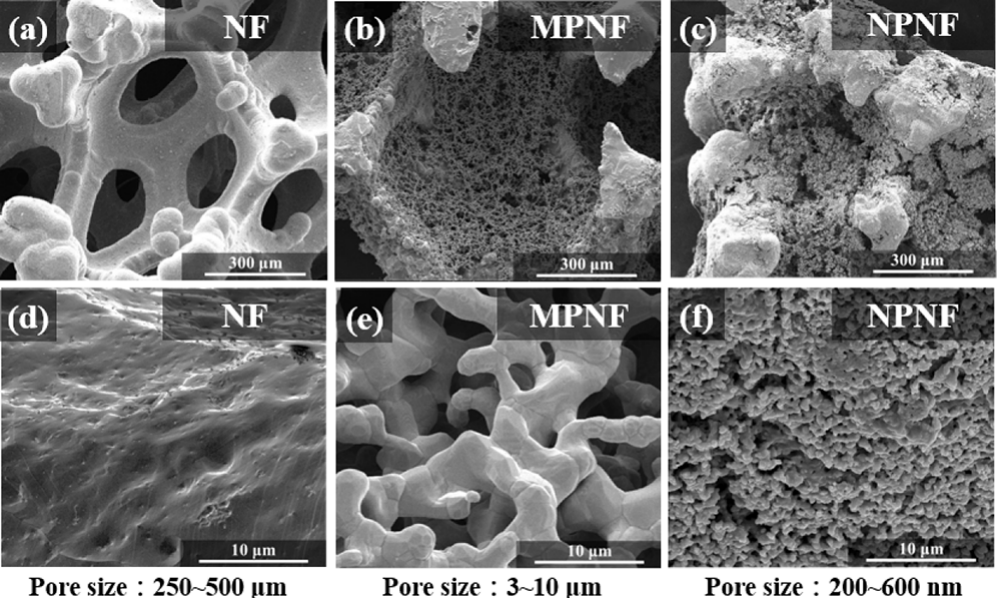
SEM images showing the (a,d) commercial NF, (b,e) MPNF, and (c,f) NPNF current collectors, respectively.

Figure 4 shows the SEM images of the commercial NF, MPNF, and NPNF current collectors at low and high magnification, respectively, revealing substantial differences in pore microstructures. As shown in Figure 4a,d, the commercial NF collector displays large pores with pore sizes ranging from 250 to 500 μm. At high magnification, the NF surface appears solid and nonporous, which limits the potential for MnO_2_ active material growth and restricts access for redox reactions. Figure 4b,e shows the MPNF current collector infused with metallic Ni powder, presenting a consistent pore size ranging approximately from 3 to 10 μm. Figure 4c,f further shows the NPNF current collector with the NiO fillers after postreduction, demonstrating the smallest pore sizes around 200–600 nm. These disparate pore sizes result from the distinctive characteristics of the pore fillers, with Ni and NiO manifesting differential behavior during sintering and reduction processes. The strong Ni–O bonding in NiO oxides impedes the diffusion of Ni atoms, decelerating the sintering rate and diminishing particle densification, consequently yielding smaller pore sizes.^46^

Hence, the integration of NiO fillers into Ni foam seamlessly aligns with the primary objective of this study, which aims to achieve finer pore sizes to bolster the specific surface area and amplify active mass loading. A suitable porosity and appropriate pore size distribution significantly contribute to superior electrochemical performance by facilitating enhanced mass transport and mitigating electrode polarization.^48^ Many studies have demonstrated that electrodes with mesopores (2–50 nm) are highly advantageous for the development of electrochemical devices.^51,53^ According to IUPAC standards, these pore sizes are classified as macropores (>50 nm). Therefore, we can further optimize the pore size distribution by adjusting the structural parameters or process integration of NiO. In addition, the nuanced comprehension of pore size and its influences on electrode performance enriches the study’s findings, providing invaluable insights into the intricate interplay among pore filler properties, sintering processes, and resultant porosity characteristics in current collectors.

**Figure 5:**
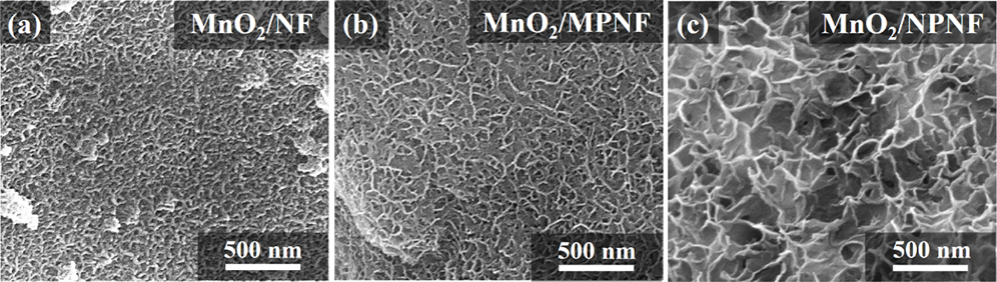
SEM images showing (a) MnO_2_/NF, (b) MnO_2_/MPNF, and (c) MnO_2_/NPNF electrodes, respectively.

Figure 5 presents the SEM images of MnO_2_ grown on NF, MPNF, and NPNF current collectors using the chemical redox process. Figure 5a shows a thin layer of MnO_2_ with small grain sizes on the surface of the commercial NF, which can be attributed to its large framework size, resulting in a limited active surface area for MnO_2_ deposition. Furthermore, Figure 5b shows the MPNF surface, which exhibits a uniform nanoflake structure of MnO_2_ due to the incorporation of Ni fillers. The incorporation of Ni fillers increases both the surface area and the deposition of active material.^50,55^Figure 5c further shows the NPNF surface with a larger, coarser MnO_2_ nanoflake structure, attributed to its nanopore structure, which provides a larger specific area for crystal growth.^56^ The chemical redox process results in mass loadings of 0.92 ^2^, 7.2, and 23.8 mg/cm^2^, for the NF, MPNF, and NPNF electrodes, respectively. These findings highlight the influence of pore filler properties and substrate morphology on MnO_2_ nanoflake growth, offering insights for optimizing the redox process to enhance electrode performance.

### 3.2 Electrochemical Performance

**Figure 6:**
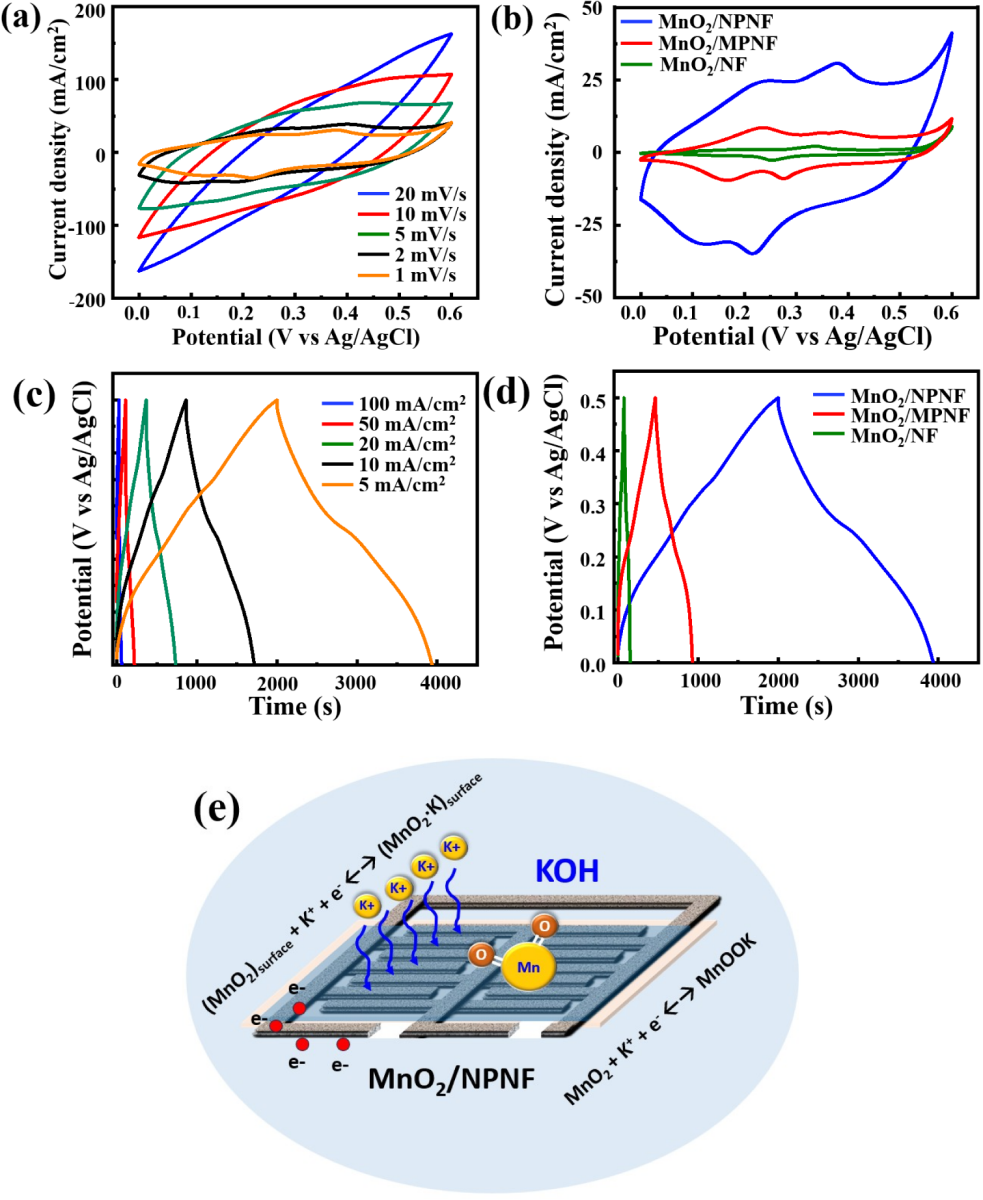
(a) CV curves of the MnO_2_/NPNF sample at different scan rates, (b) comparative CV for the MnO_2_/NF, MnO_2_/MPNF, and MnO_2_/NPNF samples at scan rate 1 mV/s, (c) GCD of the MnO_2_/NPNF samples at different current densities, (d) comparative GCD for the MnO_2_/NF, MnO_2_/MPNF, and MnO_2_/NPNF samples at current density 5 mA/cm^2^, and (e) schematic of the charge transfer process on MnO_2_/NPNF structures.

Figure 6a depicts the CV curves of the MnO_2_/NPNF electrode across various scan rates. The curve exhibits a quasi-rectangular shape at higher scanning rates, suggesting ion adsorption and desorption at the electrode/electrolyte interface, characteristic of an electric double-layer behavior.^34^ Conversely, at lower scanning rates, a pair of low redox peaks emerges, indicating the presence of a Faradaic pseudocapacitance charge storage mechanism.^57^ The mechanism of energy storage by MnO_2_ materials generally occurs through two types of processes. The first is surface adsorption–desorption, where H^+^ or basic cations (Li^+^, Na^+^, and K^+^) adsorb at the surface of the electrode to store charges. The second is electron–proton transfer, where charges are stored via fast and reversible redox reactions. In this process, H^+^ or basic cations (Li^+^, Na^+^, K^+^) intercalate into or deintercalate out of both the surface materials and the materials inside the crystal structure. The reaction equations are as follows:^58^
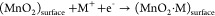
5
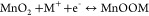
6where M^+^ represents the cation (Li^+^, Na^+^, or K^+^).

According to the literature,^59^ the appearance of a minor peak in MnO_2_ active material is closely linked to the tunnel storage mechanism. This mechanism involves one-dimensional (1D) α-MnO2 with tunnel sizes of about 4.6 Å and two-dimensional (2D) δ-MnO_2_ with tunnel sizes of approximately 7 Å. These tunnels facilitate the rapid insertion/extraction of hydrated K^+^ cations (1.13 Å) or Na^+^ cations (0.95 Å in radii) into the MnO_2_ lattice for efficient charge storage.^58,59^ The observation of the redox peak in the CV curves depends on the slow rate of the redox reaction, allowing sufficient time for electrolyte ions to diffuse through the pores.^37,60^ Noteworthy insights from the CV curve include the correlation between increased scan rates and an amplified area under the curve, indicating improved charge storage capabilities.^26,58^ Furthermore, escalating scan rates induce changes in the kinetics of electrochemical reactions, resulting in shifts in peak current density.^61^

Figure 6b summarizes a comparative analysis of CV curves among three NF-based electrodes, all acquired at a scan rate of 1 mV/s. This comparison distinctly underscores the notable superiority of the MnO_2_/NPNF electrode, which is evident from its significantly larger CV curve area. SEM analysis validates the interconnected porous nature and fine pore structure of the NPNF current collector, ranging approximately between 200 and 600 nm. Such a structural design offers a high specific surface area, as illustrated in Figure 6e, facilitating efficient charge transfer and mass transport processes.^43,60^ Furthermore, SEM examinations confirm the nanoflake morphology of MnO_2_, consistent with several earlier studies that highlight its ability to provide an extensive electrode/electrolyte contact area.^62^ Complementary BET tests, outlined in Table S1, reinforce these findings by demonstrating the substantial surface area of the MnO_2_/NPNF electrode. According to this analysis, the MnO_2_/NPNF electrode boasts a surface area of approximately 17.6 m^2^/g, about 30 times larger than that of the MnO_2_/NF electrode.

In Figure 6c, GCD tests for the MnO_2_/NPNF electrode with varying current densities (5–100 mA/cm^2^)) are illustrated. The nearly linear behavior in the charge–discharge curve signifies pseudocapacitive behavior, driven by a reversible charge storage mechanism involving oxidation and reduction reactions at the electrode–electrolyte interface.^57^ The symmetric charge–discharge curve, free from an IR drop, underscores the high Coulombic efficiency exhibited by the MnO_2_/NPNF electrodes.^63^ However, at higher current densities, the area capacitance decreases due to limited electrolyte ion accommodation within the electrode’s inner spaces, resulting in diminished capacitance values.^48^Figure 6d further compares the GCD curves for NF-based electrodes at a current density of 5 mA/cm^2^, highlighting the exceptional area coverage of the MnO_2_/NPNF electrode, indicating superior energy storage properties. Notably, the MnO_2_/NPNF electrode demonstrates a significantly prolonged discharge time of approximately 2000 s, surpassing that of the other electrodes with discharge times below 100 s. This performance trend is reflected in the area capacitance values, with MnO_2_/NPNF achieving an impressive area capacitance of 19.34 F/cm^2^, outperforming MnO_2_/MPNF (4.64 F/cm^2^) and MnO_2_/NF (0.75 F/cm^2^). These results are consistent with the mass loading of the MnO_2_ active material on these electrodes. Table 1 summarizes a comprehensive overview of the areal capacitances exhibited by MnO_2_/NF, MnO_2_/MPNF, and MnO_2_/NPNF electrodes at various current densities.

**Table 1:** Areal Capacitances of MnO_2_/NF, MnO_2_/MPNF, and MnO_2_/NPNF Electrodes at Different Current Densities

	areal capacitance (F/cm^2^)		
current density (mA/cm^2^)	MnO_2_/NPNF (23.8 mg/cm^2^)	MnO_2_/MPNF (7.2 mg/cm^2^)	MnO_2_/NF (0.92 mg/cm^2^)
100	5.80	1.14	0.18
50	10.80	2.80	0.37
20	14.68	3.68	0.52
10	17.02	4.12	0.66
5	19.34	4.64	0.75

Furthermore, several additional tests were performed to study the influences of all NF-based current collectors on capacitance values. These tests revealed battery-type behavior with a distinctive redox peak characteristic for all NF-based current collectors,^34,57^ as shown in Figure S3. However, the calculation of capacitance areas for NF and NPNF current collectors (refer to Table S2) indicates that the NF-based current collector does not significantly contribute to capacitance. Given that MnO_2_ forms only an extremely thin layer, this finding eliminates any concerns about the influence of current collectors on electrochemical performance.

**Figure 7:**
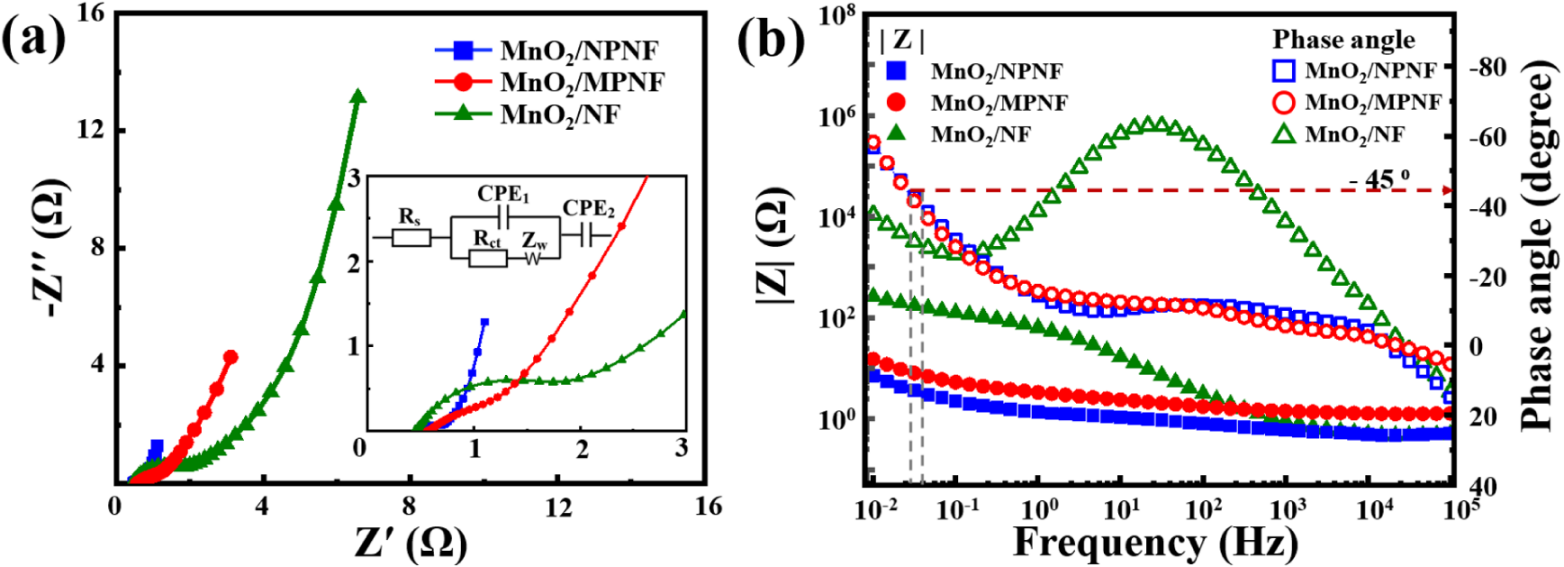
(a) Nyquist and (b) Bode plots for the EIS data.

Figure 7a presents the Nyquist plots obtained from the EIS measurements conducted over a frequency range of 0.1 Hz to 100 kHz. The MnO_2_/MNPF and MnO_2_/NPNF electrodes exhibit a small semicircular region at high frequencies, indicating a low faradaic resistance within the electrolyte. At low frequencies, the impedance path forms a high-angle straight line, which signifies the capacitive nature of MnO_2_.^59,64^ On the other hand, the MnO_2_/NF electrodes display a 45-degree impedance plot at low frequencies, suggesting the presence of Warburg impedance due to interaction with electrolyte ions in the porous electrodes.^60,64^ The inset in Figure 7a shows the equivalent circuit used to analyze resistance variations. The intercept on the real axis corresponds to the solution resistance (*R*_s_), which encompasses the intrinsic resistance of the active material and the ionic resistance of the electrolyte. All three electrodes exhibit a small *R*_s_ value (0.4–0.6 Ω), indicating minimal contact and electrolyte resistance. The semicircle diameter represents the charge transfer resistance (*R*_ct_), with the MnO_2_/NPNF electrodes showing the lowest value of 0.219 Ω, demonstrating the effectiveness of this unique structure in facilitating charge transfer at the electrode–electrolyte interface.^60^ Additionally, constant phase elements (CPE_1_ and CPE_2_) represent interfacial capacitance and pseudocapacitance, while *Z*_w_ signifies counterion diffusion resistance.

Figure 7b presents the Bode plots of all NF-based electrodes, highlighting a negative phase angle at low frequencies. Among the electrodes, MnO_2_/NPNF exhibits the highest phase angle (59°), indicating superior supercapacitor characteristics. The deviation from the ideal capacitor phase angle (90°) is attributed to the pseudocapacitive behavior of MnO_2_, which is dominated by the intercalation and deintercalation process of K^+^ ions within the MnO_2_ lattice.^65,66^ The knee frequency (*f*_0_), defined at a 45° phase angle, signifies the point at which capacitive and resistive impedances are equal. Beyond this frequency, supercapacitors demonstrate increased resistive behavior.^66^ The relaxation times (τ_0_ = *f*_0_^–1^) for the MnO_2_/NPNF and MnO_2_/MPNF electrodes are 46.5 and 21.6 s, respectively, indicating slower electrochemical processes in pseudocapacitive materials compared to traditional electric double-layer capacitors (EDLCs).^67^ Additionally, in the high-frequency region, the phase angle approaches zero, reflecting the behavior of a pure resistor.^65^ The modulus of impedance versus frequency plots reveal that the MnO_2/_NPNF electrode exhibits the lowest impedance, indicating the most favorable capacitive behavior.

**Figure 8:**
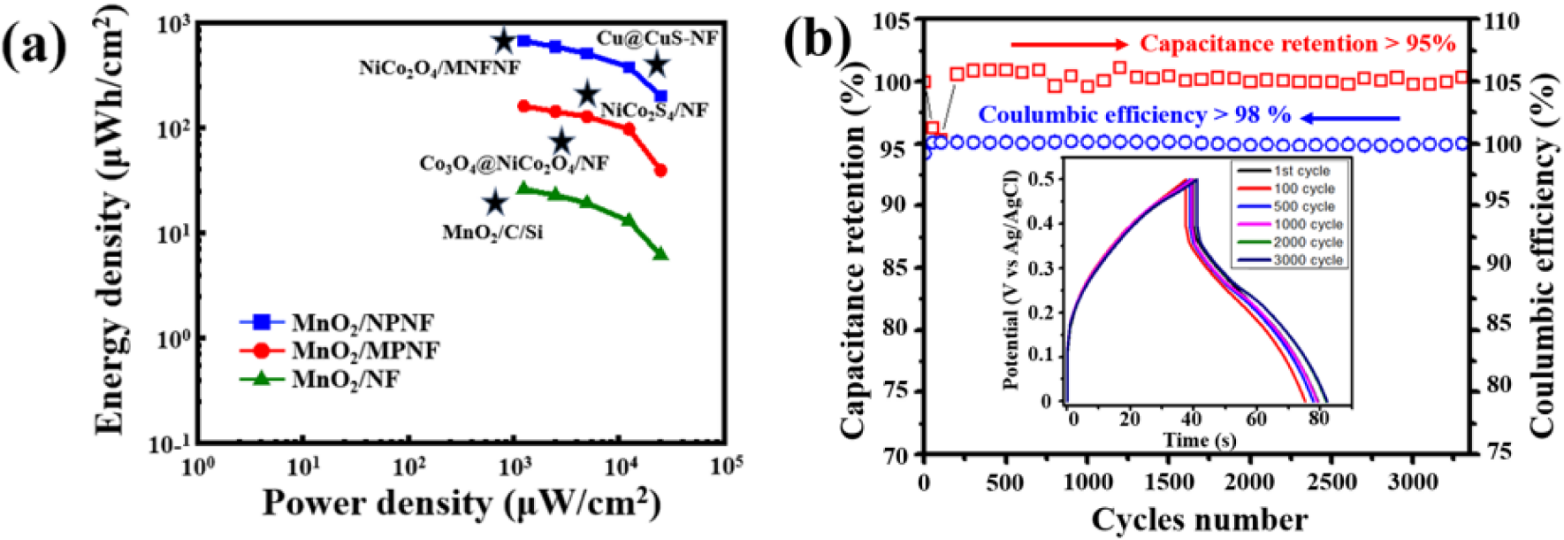
(a) Ragone plot compares the MnO_2_/NPNF, MnO_2_/MPNF, and MnO_2_/NF electrodes with the reported NF-based electrodes (shown as star mark). (b) Plots of capacitance retention, Coulombic efficiency, and CV curves vs cycle number for the MnO_2_/NPNF electrode.

In Figure 8a, the Ragone plot provides a comprehensive comparison of the energy and power densities of MnO_2_/NF-based electrodes with those of previous NF-based supercapacitor research. Notably, the energy density of MnO_2_/NF closely aligns with that of MnO_2_/C/Si.^37^ The MnO_2_/MPNF electrode demonstrates a commendable energy density, surpassing that of Co_3_O_4_@NiCo_2_O_4_/NF,^68^ though it remains below that of NiCo_2_S_4_/NF.^35^ Remarkably, the MnO_2_/NPNF electrode exhibits a peak energy density of 671 μWh/cm^2^ at a power density of 1.25 mW/cm^2^, comparable to that of Cu@CuS-NF^39^ and NiCo_2_O_4_/MNFNF.^40^ To further validate these findings, the performance of the MnO_2_/NPNF electrode was evaluated through continuous GCD testing at 5 mA/cm^2^ over 3000 cycles, as shown in Figure 8b. The MnO_2_/NPNF electrode exhibits capacitance retention exceeding 95% and Coulombic efficiency higher than 98% after 3000 cycles, indicating excellent stability. This stability underscores the robust deposition of MnO_2_ nanoflakes on the Ni foam, which remains undamaged through repeated redox cycles. In conclusion, the MnO_2_/NPNF electrode, utilizing a Ni-based current collector, emerges as a highly promising material for MSC applications based on its superior energy density and excellent long-term stability. Overall, Table S3 provides a comparison of MnO_2_/NPNF electrodes with the previous study on supercapacitor application.

### 3.3 Electrochemical Testing for Solid-State MSC Applications

**Figure 9:**
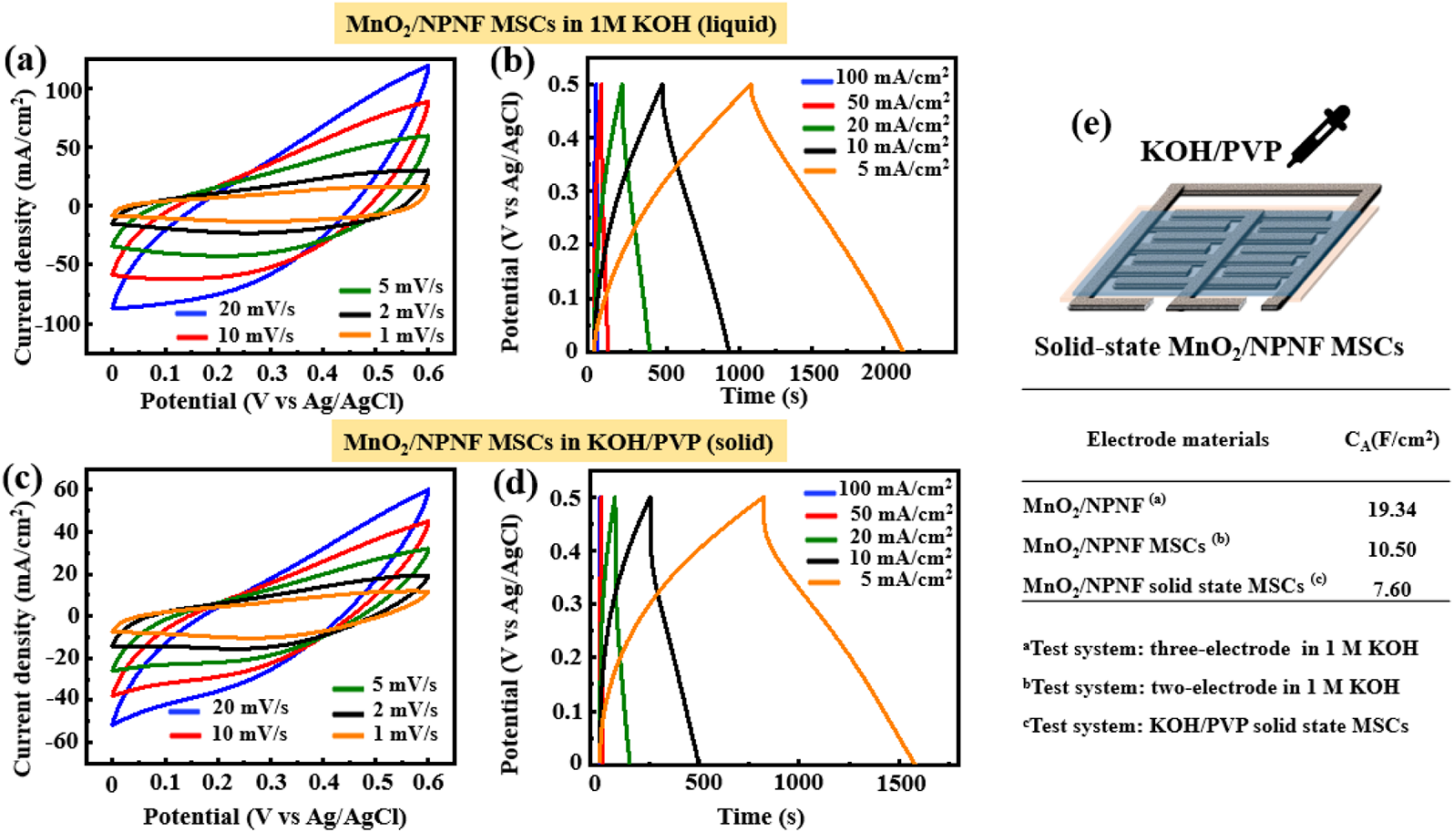
CV and GCD tests for the MSC prototypes consisting of the MnO_2_/NPNF two-electrode configuration using (a,b) 1 M KOH solution and (c,d) solid-state KOH/PVP as electrolytes. (e) Schematic of a solid-state MnO_2_/NPNF MSC and summary of electrochemical performance of different MSC devices.

Figure 9 shows the CV and GCD curves for an MSC prototype consisting of the MnO_2_/NPNF two-electrode configuration using a 1 M KOH solution as electrolyte. The CV tests performed at varying scan rates (1–20 mV/s), reveal a consistent response characterized by a quasi-rectangular shape, indicative of pseudocapacitive behavior.^58^ Compared to the three-electrode configuration, the two-electrode CV exhibits a smoother quasi-rectangular shape with lower peak current density, signifying domination by ion adsorption and desorption on the electrode surface.^14,41^Figure 9b further shows the GCD curves at different current densities, portraying a quasi-linear response with a symmetric charge–discharge region, denoting efficient charge–discharge processes.^57^ Despite a shorter discharge time and a capacitance area of 8.74 F/cm^2^ with an energy density of 364.6 μWh/cm^2^ (half that of the three-electrode system), the two-electrode MSC with MnO_2_/NPNF electrodes demonstrates a promising electrochemical performance. The observed differences may result from the absence of an auxiliary electrode as a reference, affecting potential stability and resulting in an apparent IR drop.^69,70^

Figure 9c,d further extends the electrochemical analysis to solid-state MSC application by incorporating a solid electrolyte composed of KOH and PVP, which covers the MnO_2_/NPNF interdigitated electrode structure. The CV curves also exhibit a quasi-rectangular shape at different scan rates, consistent with the previous liquid-electrolyte test, highlighting the pseudocapacitive behavior. Increased scan rates result in higher peak current density with good reversibility, affirming the stability of the MnO_2_/NPNF electrode in solid-state MSC applications. The GCD curves in Figure 9d reveal a quasi-linear response with a greater peak IR drop compared to the liquid-electrolyte test, which can be attributed to changes in ion mobility as the electrolyte changes from liquid to solid state.^69,71^ This change slows ion transport, affecting the charge–discharge process. Despite the increased IR drop, the MnO_2_/NPNF electrode demonstrates an areal capacitance value of 7.22 F/cm^2^ and an energy density of 263.9 μWh/cm^2^, indicating its potential for solid-state MSCs.^20,72^ Furthermore, the Coulombic efficiency of the MSC with the polymer gel electrolyte is estimated to be approximately 85%. Figure 9e summarizes the electrochemical performances of different MSC devices with MnO_2_/NPNF electrodes. In summary, the MnO_2_/NPNF electrode emerges as a highly promising material for solid-state MSC applications, based on its superior energy density and excellent long-term stability.

## 4 Conclusion

In conclusion, this study successfully develops a streamlined method for producing a highly nanoporous current collector with a substantial specific surface area, serving as an electrode for MSCs. Such a three-dimensional, highly nanoporous electrode dramatically increases the specific surface area by 30 times and substantially boosts the amount of MnO_2_ deposition, surpassing the capacities of commercially available Ni foams. The electrochemical analysis of the MnO_2_/NPNF electrode revealed an impressive areal capacitance of 19.3 F/cm^2^ at a current density of 5 mA/cm^2^, accompanied by an energy density of 671 μW h/cm^2^, 25 times greater than that of commercial Ni foams. Moreover, in the realm of solid-state applications for MSCs, the MnO_2_/NPNF electrode achieves a commendable areal capacity of 7.22 F/cm^2^ and an energy density of 263.9 μW h/cm^2^, rendering it exceptionally suitable for use in solid-state MSC applications. This study highlights the potential of producing a high-performance current collector through a simple and efficient technique, showing its promise in advancing the field of MSC applications.

## Supporting Information Available

(PDF) The Supporting Information is available free of charge at https://pubs.acs.org/doi/10.1021/acsomega.4c05514.

Raman spectra of MnO_2_/NF-based electrodes; BET test results and surface area plots of MnO_2_/NPNF and MnO_2_/NF electrodes; discussion about CV and GCD behaviors of bare NF-based electrodes (without MnO_2_); the areal capacitances of NF and NPNF current collectors at different current densities; performance comparison of supercapacitors with similar NF-based electrodes reported in the literature (PDF)
