# Supplementary material for: Highly Nanoporous Nickel Foam as Current Collectors in 3D All-Solid-State Microsupercapacitors
Source: ACS Omega. 2024 Aug 20;9(35):37355–64. doi: 10.1021/acsomega.4c05514 (PMC11375808; doi:10.1021/acsomega.4c05514)
Supplement: Supplementary file 1 — ao4c05514_si_001.pdf [file ao4c05514_si_001.pdf]

Supporting Information

Highly nanoporous nickel foam as current collectors in 3D all-solid-state micro-supercapacitors

Bayu Satriya Wardhana[a,b], Kuan-Wen Wang[a], Wei-Hsuan Hung[a,c], I-Yu Tsao[a], Pin-Ching Chen[a], Jason Shian-Ching Jang [a,c]*, Shih-Chieh Hsu[d]*, Sheng-Wei Lee[a,e,f]*

[a]*Institute of Materials Science and Engineering, National Central University, Taoyuan City, 32001 Taiwan, ROC*

[b]*Department of Mechanical Engineering, Brawijaya University, Malang City, 65145, Indonesia*

[c]*Department of Mechanical Engineering, National Central University, Taoyuan City, 32001 Taiwan, ROC*

[d]*Department of Chemical and Materials Engineering, Tamkang University, New Taipei City, 25137 Taiwan, ROC*

[e]*Department of Chemical and Materials Engineering, National Central University, Taoyuan City, 32001 Taiwan, ROC*

[f]*Graduate College of Sustainability and Green Energy, National Central University, Taoyuan City, 32001 Taiwan, ROC*

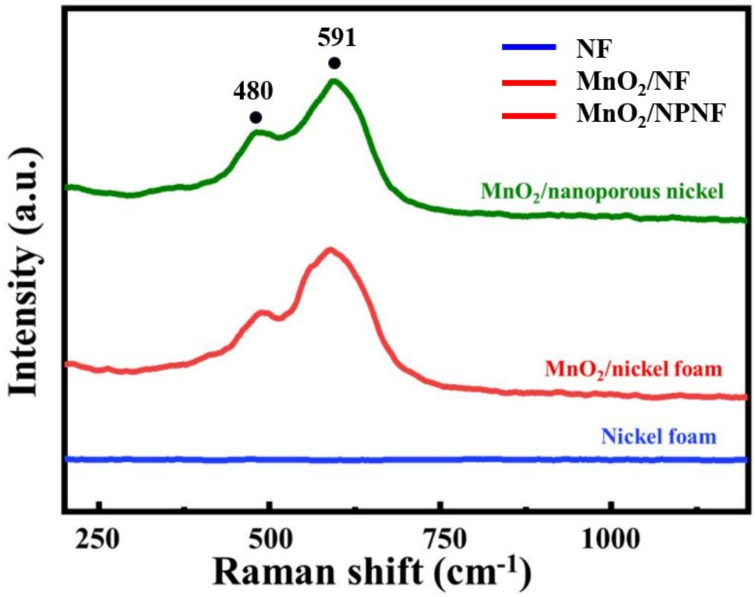

Figure S1. Raman spectra of MnO_2/NF-based electrodes in this study.

Table S1. The BET test results of MnO_2/NPNF and MnO_2/NF electrodes.

MnO_2/NPNF

BET Report

BET surface area: 17.6184 ± 0.1091 m²/g
Slope: 0.247297 ± 0.001487 g/cm³ STP
Y-intercept: -0.000249 ± 0.000359 g/cm³ STP
C: -991.915699
Qm: 4.0478 cm³/g STP
Correlation coefficient: 0.9999277
Molecular cross-sectional area: 0.1620 nm²

Relative Pressure (p/p°)	Quantity Adsorbed (cm³/g STP)	1/[Q(p°/p - 1)]
0.175294633	4.9128	0.043265
0.200288204	5.0851	0.049251
0.225363769	5.2552	0.055360
0.250520858	5.4299	0.061559
0.275589901	5.6062	0.067860
0.300666791	5.7879	0.074282

MnO_2/NF

BET Report

BET surface area: 0.5632 ± 0.0025 m²/g
Slope: 7.738316 ± 0.034054 g/cm³ STP
Y-intercept: -0.010665 ± 0.005256 g/cm³ STP
C: -724.564539
Qm: 0.1294 cm³/g STP
Correlation coefficient: 0.9999710
Molecular cross-sectional area: 0.1620 nm²

Relative Pressure (p/p°)	Quantity Adsorbed (cm³/g STP)	1/[Q(p°/p - 1)]
0.100196456	0.1452	0.766779
0.125351165	0.1496	0.958041
0.149940992	0.1536	1.148709
0.175435851	0.1583	1.344251
0.200186633	0.1624	1.541228

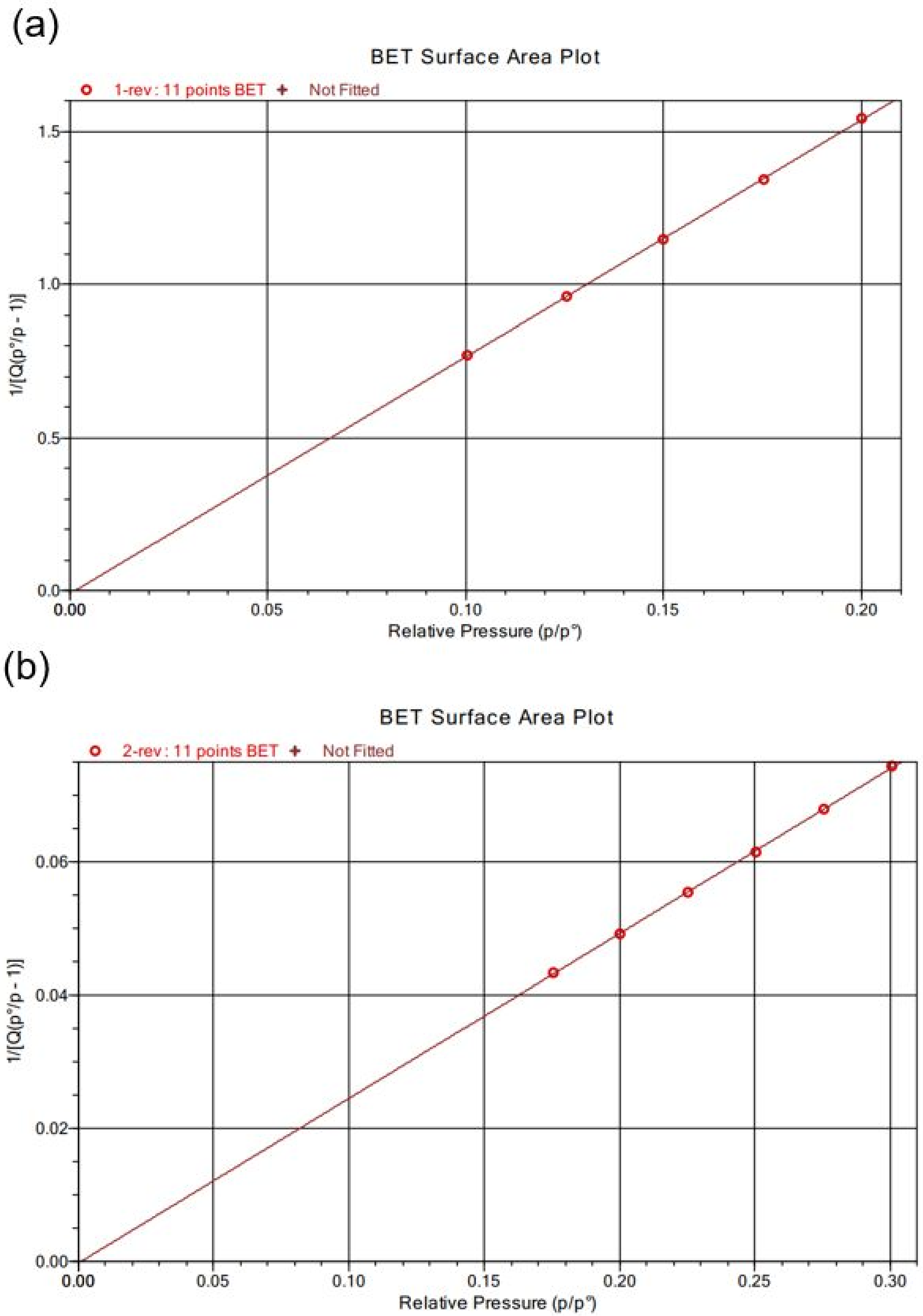

Figure S2. BET surface area plots of the (a) MnO_2/NF and (b) MnO_2/NPNF electrodes.

Figure S3(a)-S3(c) shows the CV curve at different scan rates (1-10 mV/s) and the GCD curve at different current densities (5-100 mA/cm^2) for the NPNF, MPNF, and NF current collector, respectively. Without the MnO_2 coating, we can see strong oxidation peaks in CV curves at various scan rates, and plateau shape GCD curves at different current densities, indicating that nickel-based current collector shows battery-type behaviors.

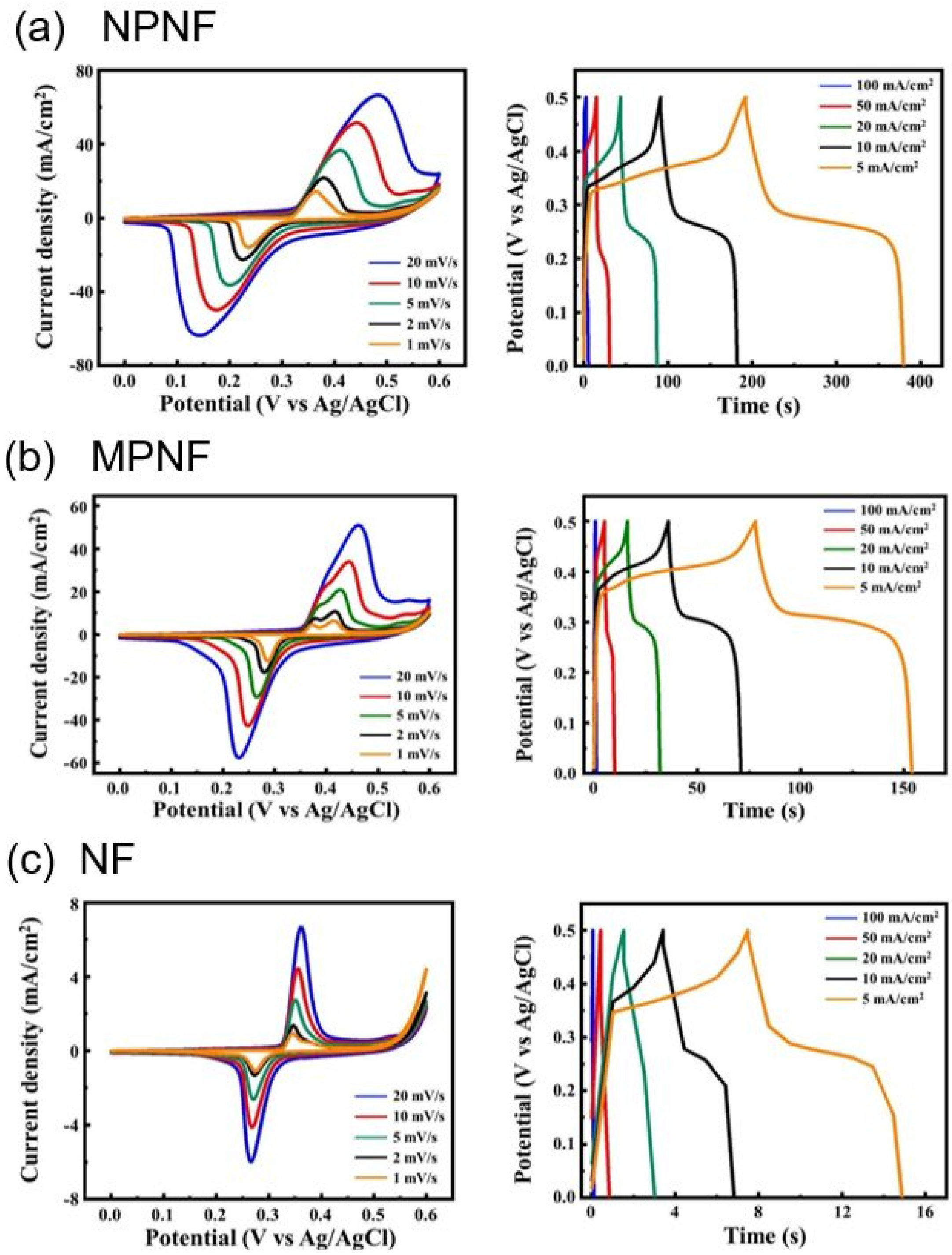

Figure S3. CV curves at different scan rates and GCD curves at different current densities of the (a) NPNF, (b) MPNF, and (c) NF current collector, respectively.

Table S2. The areal capacitances of NF and NPNF current collectors at different current densities.

Current Density (mA/cm^2)	Areal Capacitance (F/cm^2)	
	NF current collector	NPNF current collector
100	9.12×10^{-3}	5.98×10^{-1}
50	4.20×10^{-2}	1.53
20	6.05×10^{-2}	1.72
10	6.80×10^{-2}	1.80
5	7.40×10^{-2}	1.87

Table S3. Performance comparison of supercapacitors with similar NF-based electrodes reported in the literature.

Electrode material	Current collector	Capacitance	Current density	Stability	Energy density	Power density	Ref. No.
$NiCo_2O_4$	MNFNF	23.5 F/cm^2	5 mA/cm^2	98% @ 1000 cycles	1,38 mWh/cm^2	12.5 mW/cm^2	40
Cu@CuS	NF	11.4 F/cm^2	90 mA/cm^2	100% @ 3000 cycles	994 $\mu Wh/cm^2$	22.5 mW/cm^2	39
$NiCo_2S_4$	NF	4000 mF/cm^2	7 mA/cm^2	89% @ 10.000 cycles	200 $\mu Wh/cm^2$	45 mW/cm^2	35
Co_3O_4@$NiCo_2O_4$	NF	2.04 F /cm^2	10 mA/cm^2	83.75% @ 7500 cycles	13.8 $\mu Wh/cm^2$	2.5 mW/cm^2	68
MnO_2/C	Si	223.74 mF/ cm^2	5 $\mu A/cm^2$	>92% @ 4000 cycles	2.62 μWh/ cm^2	117.8 μW/ cm^2	37
MnO_2	NF	19.3 F/cm^2	5 mA/cm^2	95% @ 3000 cycles	671 $\mu W h/cm^2$	1.25 mW/cm^2	This work
